# Transactions of the American Dental Association, 1880

**Published:** 1881-02

**Authors:** 


*Transactions of the American Dental Association*, at the Twentieth Annual Session, held at Boston, commencing August 3d, 1880. Publishers: Trustees of S. S. White, 1881.

Publication Committee, Drs. Geo. H. Cushing, M. S. Dean and E. T. Darby, to whom great credit is due for the arrangement of a very interesting Report of Proceedings, which contains much valuable information.

